# Adaptable probabilistic mapping of short reads using position specific scoring matrices

**DOI:** 10.1186/1471-2105-15-100

**Published:** 2014-04-09

**Authors:** Peter Kerpedjiev, Jes Frellsen, Stinus Lindgreen, Anders Krogh

**Affiliations:** 1 Section for Computational and RNA Biology, Department of Biology, University of Copenhagen, Ole Maaloes Vej 5, 2200 Copenhagen, Denmark; 2 Institute for Theoretical Chemistry, University of Vienna, Währinger Straße 17, A-1090 Vienna, Austria; 3 Department of Engineering, University of Cambridge, Trumpington Street, Cambridge CB2 1PZ, UK; 4 Biomolecular Interaction Centre, School of Biological Sciences, University of Canterbury, Private Bag 4800, Christchurch 8020, New Zealand; 5 Centre for GeoGenetics, Natural History Museum of Denmark, University of Copenhagen, Øster Voldgade 5-7, 1350 Copenhagen K, Denmark

**Keywords:** Short-read mapping, Sequence alignment, Next-generation sequencing, Ancient DNA, PAR-CLIP, Xeno mapping

## Abstract

**Background:**

Modern DNA sequencing methods produce vast amounts of data that often requires mapping to a reference genome. Most existing programs use the number of mismatches between the read and the genome as a measure of quality. This approach is without a statistical foundation and can for some data types result in many wrongly mapped reads. Here we present a probabilistic mapping method based on position-specific scoring matrices, which can take into account not only the quality scores of the reads but also user-specified models of evolution and data-specific biases.

**Results:**

We show how evolution, data-specific biases, and sequencing errors are naturally dealt with probabilistically. Our method achieves better results than Bowtie and BWA on simulated and real ancient and PAR-CLIP reads, as well as on simulated reads from the AT rich organism *P. falciparum*, when modeling the biases of these data. For simulated Illumina reads, the method has consistently higher sensitivity for both single-end and paired-end data. We also show that our probabilistic approach can limit the problem of random matches from short reads of contamination and that it improves the mapping of real reads from one organism (*D. melanogaster*) to a related genome (*D. simulans*).

**Conclusion:**

The presented work is an implementation of a novel approach to short read mapping where quality scores, prior mismatch probabilities and mapping qualities are handled in a statistically sound manner. The resulting implementation provides not only a tool for biologists working with low quality and/or biased sequencing data but also a demonstration of the feasibility of using a probability based alignment method on real and simulated data sets.

## Background

Next-generation DNA sequencing is a powerful tool in biological research [1] and is steadily gaining momentum as costs keep decreasing. Applications vary from genome re-sequencing [2-4] to transcriptome analysis [5-7], metagenomics projects [8-10], and sequencing of ancient genomes [11-16]. All these applications rely on mapping reads to existing reference genomes. Many mapping programs have been developed using a variety of algorithms with different strengths, weaknesses and limitations [17].

Hash-based algorithms such as MAQ [18] and SOAP [19] dominated initially but were hampered by large memory demands. Subsequently, the Burrows-Wheeler transform [20] was applied to compress the genome index in programs such as Bowtie [21], Bowtie2 [22], SOAP2 [23], and BWA [24]. This decreased the memory usage while increasing speed and sensitivity, leading to mappers based on the Burrows-Wheeler transform to now dominate the field. Other approaches, however, show promising results for some types of data. Segemehl [25] uses an enhanced-suffix array to provide fast alignment of insertion/deletion (indel) prone reads, and a similar approach was implemented in the mapping tool used in the sequencing of the first ancient human genome [13]. Programs such as CUSHAW [26] and SOAP3 [27] have begun to use graphics processing units (GPUs) to provide even faster mapping.

Most programs allow a specified number of mismatches in an alignment (with a limit of 1-3 mismatches in the beginning of the read), and report uniquely mapped reads as those where all other locations have more mismatches. However, evaluating a mapping location by the number of nucleotide mismatches alone is not optimal and implicitly assumes that the genome has a homogeneous base composition and that errors occur uniformly in the reads. The mapping with the lowest number of mismatches may have a high probability of being incorrect if i) there are many sub-optimal mappings, ii) the genome has a very biased base composition, iii) certain nucleotide mismatches are expected due to sample conditions, or iv) the mismatching bases have low error-probabilities compared to other bases.

Most sequencing platforms provide a quality score for each base derived from the probability that the nucleotide is wrongly assigned in the base-calling. With the Illumina platform, the error probabilities typically range from around 0.01% in the 5’ end of the read to several percent in the 3’ end, but the actual DNA sequence can affect the read quality [28]. These qualities can affect not only the ability of a mapper to find the correct hit, but also the quality of the reported hit. While the latest generation of mappers such as MASAI [29] and GEM [30] either do not take quality scores into account or only consider them in a rudimentary manner, they also report all possible alignments and do not provide a mapping quality to distinguish between a high confidence alignment and a low confidence one. We demonstrate that taking quality scores and other information about the biases in the experimental data into account can improve the sensitivity while providing an accurate mapping quality estimation. Recently, the use of position-specific scoring matrices (PSSMs) has been applied to the short read mapping problem and shown to provide accurate SNP and indel calling [17,31].

Here we show how quality scores, contamination, biases in base composition, mutations, and data-specific base-changes (as in PAR-CLIP or ancient DNA) can all be dealt with probabilistically and encoded in a PSSM. We also present an algorithm which applies the Burrows-Wheeler transform to the scoring of PSSMs, and BWA-PSSM, a fast and sensitive mapping tool which implements the methods.

We show that the use of probabilistic scoring allows for both higher sensitivity and positive predictive value when mapping simulated reads. More importantly, we offer the ability to use a specified error model to map reads based on the expected types and locations of mismatches. This improves the mapping of ancient DNA data with errors due to damage, and the improvement is even more pronounced when mapping PAR-CLIP data, where there is a strong bias towards T-to-C substitutions [32], as well as data from *P. falciparum* which has an extreme nucleotide bias with more than 80% AT content [33]. We also show that the probabilistic scoring can help in cases of contamination by dramatically reducing the mapping of short reads from *E. coli* to the human genome.

## Results and discussion

When mapping reads, one is interested in the probability that the read originated from a specific location in the genome. The read and the genome might not be completely identical due to e.g. sequencing errors or SNPs.

### A position-specific scoring matrix from quality scores

Most sequencing machines provide a quality score for each base which is related to the probability of a sequencing error occurring at this position in the read. These qualities are normally in the Phred format [34] and relate the error probability *p*_e_ and the quality score *Q* by *p*_e_ = 10^-*Q*/10^. From these qualities, we can calculate the probability *P*(*a*|*x*) for the base *a* ∈ {A,C,G,T} being present at a given position in the DNA fragment given that the base *x* ∈ {A,C,G,T} was called by the sequencing machine (see Methods).

If the DNA being sequenced differs from the reference genome, e.g. due to evolution, there is a probability *P*(*g*|*a*) that base *g* occurs in the genome if the base is *a* in the sample. We use a simple model with a probability *p*_0_ for a mutation (see Methods).

Combining this with the probability of errors, the probability of a base *g* in the genome given the called base *x* is

(1) $$P(g|x)=\underset{a}{\sum }P(g|a)P(a|x).$$

In some types of data, known base modifications are known to occur. For instance, in ancient DNA some bases are damaged due to hydrolysis, resulting in cytosine to thymine (C-to-T) conversions in the 5’ ends of reads, which can look like apparent guanine to adenine (G-to-A) substitutions in the 3’ end, and in PAR-CLIP experiments there is a large fraction of thymine to cytosine (T-to-C) conversions where the protein crosslinks to the RNA. These phenomena are easily modeled and included in the PSSM, essentially by introducing a probability *P*(*b*|*a*) that the observed base is *b*, given that the base is *a* in the sample (see Methods section).

Using this approach, one can turn a short read into a PSSM and use it for mapping the read to the genome. The PSSM is constructed such that at position *i* in the read, the score for base *g* is *s*(*g*|*x*_*i*_) = log_2_(*P*(*g*|*x*_*i*_)/*q*(*g*)), where *P*(*g*|*x*_*i*_) is the probability of a genomic base *g* given the *i*’th base *x*_*i*_ in the read calculated as described above in equation 1, and *q*(*g*) is a background probability, which is usually just the frequency of the base in the genome. The score for matching a read **x** to a certain position *ℓ* in the genome is *S*_**x**_(*ℓ*), and is obtained by summing the scores from the PSSM corresponding to the genome sequence starting at position *l*.

BWA-PSSM is very flexible when it comes to how you feed the PSSM to the program. In the present implementation, the PSSM can be constructed from the quality scores supplied with the sequence, using a background distribution calculated from the frequencies of bases in the reference genome and a mutation rate (*p*_0_ above). Alternatively, the user can supply a table with a direct translation of base and quality score pairs to PSSM scores. Such a table can be computed ahead of time to include for instance a more sophisticated evolutionary model or a PAR-CLIP model. Finally the user can input a fully constructed PSSM and use it for mapping.

### Mapping probability

The advantage of the PSSM is that the probability of a match can be calculated directly [17]. There is a probability that a read **x** originates from – or is homologous to – a position *ℓ* in a genome **g**, *P*(*ℓ*,M|**x**,**g**), where M means the "match model". Alternatively, the background model N is used if the read does not originate from the genome due to e.g. contamination or adapter sequences. *A priori*, we may be able to estimate the probability *P*(N|**x**) of a read being contamination, and obviously *P*(M|**x**) = 1 - *P*(N|**x**).

Using the sum and product rule we can express the match probability at position *ℓ* as

$$P(\ell ,M|x,g)=\frac{P(g|\ell ,M,x)P(\ell |M,x)P(M|x)}{P(g|M,x)P(M|x)+P(g|N,x)P(N|x)}.$$

Assuming that any mapping position is equally likely *a priori* we have *P*(*ℓ*|M,**x**) = 1/*L*, where *L* is the length of the genome.

In the background model N we assume independently identically distributed (i.i.d.) bases.

In the match model M, the probability of the aligned bases in **g** is calculated from the PSSM and the remaining bases are assumed to be distributed as in the background model; this means that

$P(g|\ell ,M,x)/P(g|N,x)=2^{S_{x}(\ell )}$, where *S*_**x**_(*ℓ*) is the PSSM score. It is possible, of course, to have more sophisticated background models of those sequences that do not originate from the genome, such as a Markov model, but this will not be considered here.

Using the identity $P(g|M,x)=\sum_{\ell^{\prime }}P(g|\ell^{\prime },M,x)P(\ell^{\prime }|M,x)$, where *ℓ*^′^ runs over all positions in the genome, we can finally write the posterior probability of the read **x** mapping to position *ℓ* in the genome as

(2) $$P(\ell ,M|x,g)=\frac{2^{S_{x}(\ell )}}{\sum_{\ell^{\prime }}2^{S_{x}(\ell^{\prime })}+L(1-P(M|x))/P(M|x)}.$$

The first term in the denominator is the sum over all possible genomic positions. This is impractical to calculate, but since only positions with some similarity to the query yield a significant contribution, it is well approximated by a sum over high-scoring mappings. The second term in the denominator is proportional to the genome size and reflects the fact that the larger the genome, the more likely it is to have a random hit.

In the BWA-PSSM implementation we assume the same prior match probability for all reads, *P*(M) = *P*(M|**x**), which can be specified as a parameter with a default value of 0.8 which is used for all experiments in this paper. To simplify the presentation we assumed no indels in the alignment of the read and the genome, see Additional file 1 for the derivation of equation (2) and Methods for details on handling indels.

### Algorithm and implementation

PSSM search is implemented in the BWA program [24] as a separate version called BWA-PSSM, which can be used instead of the regular BWA alignment step (aln). Here we describe the main algorithmic changes as compared to standard BWA.

Using a PSSM, a score can be calculated for each position in the reference genome, where high scores indicate hits. Using a Burrows-Wheeler transformed index, this operation can be sped up by evaluating scores on the prefix tree of the index. At any given point in the search, the position within the scoring matrix corresponds to the current depth of the prefix tree. Scores are calculated by summing the position-specific score at each node along a path.

To bound the number of positions in the genome that must be evaluated, a threshold score is used which replaces the maximum number of mismatches used in standard BWA. To calculate an overall score threshold for a read, we rely on converting a limit on the number of mismatches *n* into the minimum possible score with *n* mismatches. That is, we allow *n* mismatches of bases with high quality and more than *n* mismatches of low-quality bases.

To further prune the search space, lookahead scoring can be used, in which the threshold is calculated for each position in the PSSM as the difference between the threshold and the best possible score of the remaining PSSM [35]. This is implemented in BWA-PSSM (Algorithm 1), but using an improved bound. This is done by adapting the method (named CALCULATED) employed by BWA to consider what the minimum number of mismatches must be for that subsequence to align to some region of the genome. Our algorithm, called CALCULATET (Algorithm 2), instead calculates the difference between the best possible PSSM score with no mismatches and the best possible score with the minimum number of necessary mismatches. This difference is calculated at each position and added to the lookahead-derived intermediate thresholds. This has the effect of requiring a higher match score at each position and thus further bounding the search tree. This allows for faster and more accurate mapping of sequences with many low quality bases as more likely paths (and thus matches) will tend to be visited first.

**Algorithm 1 The recursive BWA-PSSM search algorithm.** The PSSM is denoted *A* and the PSSM thresholds at each position*i* are stored in *T*[*i*]. A score, *s*, is maintained for every partial match and the indices into the Burrows-Wheeler transformed sequence are stored in *k* and *l*. In the algorithm *O*(*b*,*l*) denotes the number of occurrences of the base *b* in the *l*’th prefix of the Burrows-Wheeler transformed reference sequence, and *C*(*b*) is the number of occurrences of bases that are lexicographically smaller than *b* in the reference sequence. Insertions and deletions are assigned penalties *ρ*_**i**_ and *ρ*_**d**_, respectively. The algorithm is initiated with the values PSSMSEARCH(*A*,*T*,|**x**|-1,0,|**g**|-1), where*T* is calculated from the sequence **x** using the algorithm CALCULATET and |**g**| denotes the size of the reference sequence.

**Algorithm 2 Calculation of intermediate thresholds.** The algorithm calculates intermediate thresholds for PSSM *A*, read sequence **x**, genomic reference sequence **g** given the global threshold *t*. **x**_***j*:*i***_ is the subsequence from *j* to *i* of the read, and *t* stores the difference between the best and second best PSSM score that can be obtained for the subsequence. The MINDROP(*A*,*i*,*j*) function simply calculates the minimum of the differences between the highest and lowest scores for columns *i* to *j* in the PSSM, while the function SUMMAX(*A*,0,*i* - 1) calculates the sum of the maximal values in column 0 to column *i* - 1 in the PSSM.

While BWA uses the MAQ mapping quality score (MapQ), BWA-PSSM reports the posterior probability given in equation (2), but estimating the sum in the denominator from the matches above the threshold. In keeping with the MAQ tradition, this probability is also log scaled, rounded to an integer and reported as the MapQ score in the output, that is $M_{Q}=\lfloor -10\log_{10}(P(l,M|x))+\frac{1}{2}\rfloor$.

### Comparing methods on simulated reads

**Figure 1:**
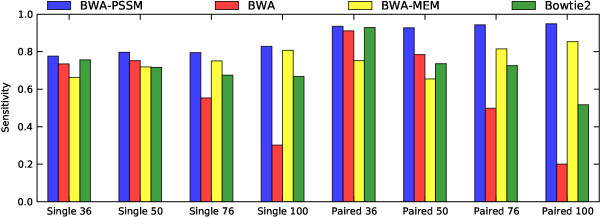
**Comparison of the sensitivity of BWA-PSSM, BWA, BWA-MEM and Bowtie2 after applying MapQ filtering on the single-end and paired-end data sets simulated with ART.** After filtering the results on mapping quality all the mappers shown above have a PPV above 0.99. The sensitivities and PPVs are listed in Tables 1 and 2. Bowtie and GEM are excluded as they do not provide MapQ scores.

**Figure 2:**
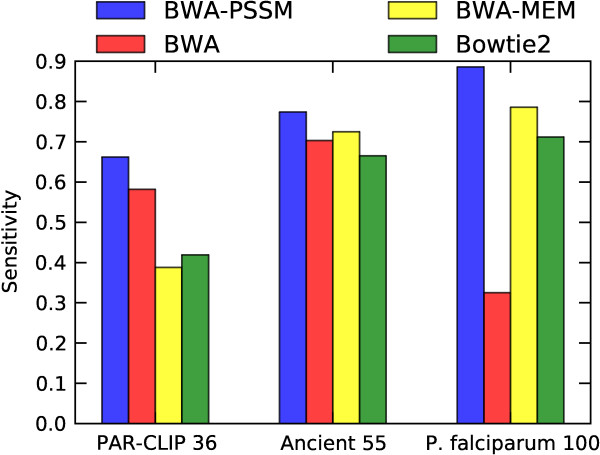
**Comparison of the sensitivity of BWA-PSSM, BWA, BWA-MEM and Bowtie2 for the three simulated biased data sets after applying MapQ filtering.** After filtering the results on mapping quality all the mappers shown above have a PPV above 0.97, except for BWA-MEM which has a PPV of 0.868 on the *P. falciparum* data set. For the PAR-CLIP data we show the sensitivity of BWA-PSSM^PC^ using the T-to-C transition model, and for the ancient DNA data the sensitivity of BWA-PSSM^A^ using the ancient DNA damage model is shown. The sensitivities and PPVs are listed in Table 3. Bowtie and GEM are excluded as they do not provide MapQ scores.

The performance of BWA-PSSM was compared to BWA [24], BWA-MEM [36], Bowtie [21], Bowtie2 [22] and GEM [30] on a number of simulated datasets modelling different types of short reads. The results are summarized in Tables 1, 2 and 3 and Figures 1 and 2, and details are given below. Each program, except Bowtie, reports a mapping quality (MapQ), and the mapping performance clearly depends on which threshold is used for this. We report the unfiltered results, for which we use no MapQ threshold, and the filtered results, for which all matches with a MapQ of less than 25 are discarded. The sensitivity reported is the number of correctly mapped reads divided by the total number of reads. The PPV (Positive Predictive Value) is the number of correctly mapped reads divided by the number of reported matches. The elapsed user time is reported when running each program on an Intel(R) Xeon(R) E7450 2.40GHz CPU.

**Table 1:** Analysis of single-end data simulated with ART

		**Unfiltered**		**MapQ filtered**		
	**Mapper**	**Sensitivity**	**PPV**	**Sensitivity**	**PPV**	**Time (s)**
**a) ART single-end length 36**						
	BWA-PSSM	0.811	0.899	0.776	1.000	44.19
	BWA	0.804	0.896	0.735	1.000	47.41
	BWA-MEM	0.772	0.900	0.663	1.000	146.99
	Bowtie	0.812	0.900	*	*	9.75
	Bowtie2	0.802	0.898	0.757	0.999	28.67
	GEM	0.755	0.995	*	*	34.57
**b) ART single-end length 50**						
	BWA-PSSM	0.839	0.934	0.797	0.999	96.07
	BWA	0.805	0.929	0.753	0.999	84.65
	BWA-MEM	0.816	0.921	0.719	1.000	56.64
	Bowtie	0.840	0.931	*	*	15.74
	Bowtie2	0.802	0.918	0.717	0.999	48.58
	GEM	0.705	0.995	*	*	35.45
**c) ART single-end length 76**						
	BWA-PSSM	0.807	0.967	0.795	0.998	94.35
	BWA	0.573	0.961	0.554	0.998	168.42
	BWA-MEM	0.821	0.937	0.751	0.999	65.21
	Bowtie	0.822	0.962	*	*	25.01
	Bowtie2	0.778	0.928	0.675	1.000	81.86
	GEM	0.695	0.995	*	*	32.20
**d) ART single-end length 100**						
	BWA-PSSM	0.837	0.979	0.828	0.999	128.25
	BWA	0.308	0.973	0.302	0.998	262.54
	BWA-MEM	0.863	0.951	0.807	0.999	91.12
	Bowtie	0.855	0.976	*	*	28.33
	Bowtie2	0.811	0.944	0.668	1.000	100.49
	GEM	0.716	0.996	*	*	35.77

Comparison of sensitivity, positive predictive value (PPV) and run time using BWA-PSSM, BWA, BWA-MEM, Bowtie, Bowtie2 and GEM on simulated data sets covering a random 1% of the human genome. The reads were simulated using the ART_illumina [37] program with the default error profile for the given read length. The symbol ’*’ indicates that the mapper does not provide MapQ scores.

**Table 2:** Analysis of paired-end data simulated with ART

		**Unfiltered**		**MapQ filtered**		
	**Mapper**	**Sensitivity**	**PPV**	**Sensitivity**	**PPV**	**Time (s)**
**a) ART paired-end length 36**						
	BWA-PSSM	0.974	0.974	0.935	1.000	211.18
	BWA	0.973	0.973	0.911	1.000	238.95
	BWA-MEM	0.899	0.899	0.753	1.000	404.35
	Bowtie	0.483	0.927	*	*	1578.74
	Bowtie2	0.971	0.971	0.929	0.999	137.88
	GEM	0.976	0.071	*	*	91.46
**b) ART paired-end length 50**						
	BWA-PSSM	0.965	0.967	0.928	0.999	242.98
	BWA	0.911	0.943	0.785	0.999	261.53
	BWA-MEM	0.902	0.908	0.655	1.000	118.35
	Bowtie	0.433	0.951	*	*	656.37
	Bowtie2	0.939	0.952	0.736	1.000	109.78
	GEM	0.927	0.399	*	*	48.79
**c) ART paired-end length 76**						
	BWA-PSSM	0.958	0.978	0.943	0.999	155.57
	BWA	0.697	0.963	0.498	0.999	210.34
	BWA-MEM	0.939	0.943	0.815	1.000	102.00
	Bowtie	0.366	0.969	*	*	213.10
	Bowtie2	0.920	0.948	0.726	1.000	81.17
	GEM	0.906	0.692	*	*	29.47
**d) ART paired-end length 100**						
	BWA-PSSM	0.962	0.983	0.949	0.999	147.38
	BWA	0.352	0.971	0.201	0.999	194.03
	BWA-MEM	0.955	0.956	0.854	1.000	102.86
	Bowtie	0.365	0.981	*	*	168.39
	Bowtie2	0.930	0.957	0.517	1.000	74.23
	GEM	0.900	0.779	*	*	23.25

Comparison of sensitivity, positive predictive value (PPV) and run time using BWA-PSSM, BWA, BWA-MEM, Bowtie, Bowtie2 and GEM on simulated data sets covering a random 1% of the human genome. The reads were simulated using the ART_illumina [37] program with the default error profile for the given read length. The insert sizes in the paired-end data were simulated using a mean length of 250 and a standard deviation of 50. The symbol ’*’ indicates that the mapper does not provide MapQ scores.

**Table 3:** Analysis of simulated biased single-end data

		**Unfiltered**		**MapQ filtered**		
	**Mapper**	**Sensitivity**	**PPV**	**Sensitivity**	**PPV**	**Time (s)**
**a) ART single-end length 36 / PAR-CLIP**						
	BWA-PSSM^PC^	0.699	0.881	0.662	0.996	68.84
	BWA-PSSM	0.642	0.865	0.594	0.990	81.89
	BWA	0.628	0.866	0.582	0.996	56.05
	BWA-MEM	0.451	0.844	0.388	0.999	74.78
	Bowtie	0.689	0.870	*	*	32.21
	Bowtie2	0.594	0.845	0.419	0.992	25.91
	GEM	0.475	0.980	*	*	39.48
**b) ART single-end length 55 / Ancient DNA**						
	BWA-PSSM^A^	0.807	0.941	0.774	0.997	122.16
	BWA-PSSM	0.797	0.937	0.766	0.996	115.17
	BWA	0.743	0.935	0.703	0.998	90.33
	BWA-MEM	0.817	0.924	0.725	1.000	50.89
	Bowtie	0.807	0.934	*	*	28.55
	Bowtie2	0.788	0.916	0.665	0.999	57.76
	GEM	0.691	0.993	*	*	30.62
**c) ART single-end length 100 /*P. falciparum***						
	BWA-PSSM	0.899	0.975	0.886	1.000	86.60
	BWA	0.332	0.974	0.325	0.999	59.20
	BWA-MEM	0.824	0.840	0.786	0.868	34.56
	Bowtie	0.925	0.976	*	*	12.91
	Bowtie2	0.832	0.972	0.712	1.000	34.33
	GEM	0.726	1.000	*	*	13.84

Comparison of sensitivity, positive predictive value (PPV) and run time using BWA-PSSM, BWA, BWA-MEM, Bowtie, Bowtie2 and GEM on data sets simulated using the ART_illumina [37] program with the default error profile for the given read length. The PAR-CLIP and Ancient DNA data sets cover a random 1% of the human genome, while the *P. falciparum* data set covers 42.9% of the *P. falciparum* genome corresponding to 100,000 reads. The data simulated for the PAR-CLIP and Ancient DNA data was further mutated to simulate the bias introduced by the experiment and natural degradation (see the respective Results and discussion sections). The symbol ’*’ indicates that the mapper does not provide MapQ scores.

#### Unbiased reads

To test the baseline performance of BWA-PSSM, we simulated reads using three different simulation programs. The first, ART [37] simulates reads using an error profile particular to the sequencing technology being simulated (Illumina Genome Analyzer II, in our case). The second, WG-SIM [38], simulates reads with a uniform error probability. The third, MASON [39], uses variable, position dependent qualities drawn from normal distributions with a specified mean and standard deviation for the start and end position. ART generated the lowest quality reads, followed by MASON, followed by the high quality WG-SIM simulated reads.

As expected, BWA-PSSM performs best on the low-quality data set (Table 1) and slightly worse on the high quality reads (Additional file 1: Table S2 and S4). It performs comparatively better on the shorter reads than on the longer. This is likely explained by the fact that we limit the size of the heap (and consequently the branching) and thus miss more hits simply because we discard a proportionally larger portion of the search space for the longer reads. Filtering according to mapping quality improves the PPV and reduces the sensitivity.

**Figure 3:**
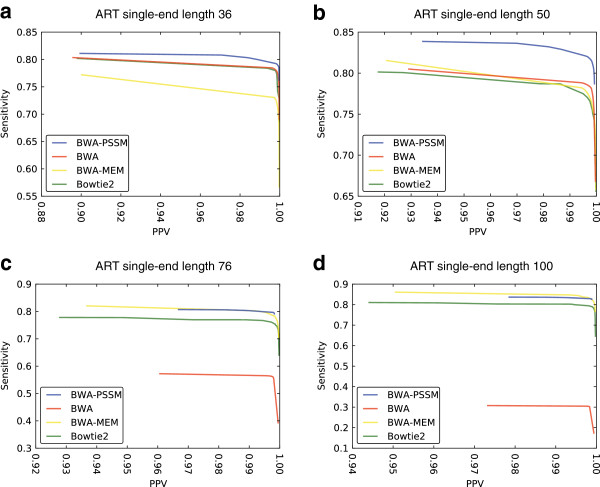
**Sensitivity as a function of PPV for BWA-PSSM, BWA, BWA-MEM and Bowtie2 using single-end ART-simulated data.** For short reads of length 36 **(a)** and 50 **(b)** BWA-PSSM shows greater sensitivity than the other mappers at similar PPV. For reads of length 76 **(c)** the performance of BWA-PSSM and BWA-MEM is similar, while for reads of length 100 **(d)** BWA-MEM has slightly higher sensitivity than BWA-PSSM at similar PPV. The curves for each mapping program were obtained by filtering for varying mapping qualities. The results are based on the simulations shown in Table 1. Bowtie and GEM are excluded as they do not provide MapQ scores.

The performance of BWA-PSSM really stands out when considering high quality alignments (as reported by the MapQ score) of ART-simulated reads (Figure 1). BWA-PSSM achieves the greatest sensitivity of the tested aligners which all have a PPV greater than 99%. For reads of length 36 and 50, BWA-PSSM performs better across all quality values, whereas for the longer reads BWA-MEM reports more lower quality mappings (Figure 3). When ignoring the quality of the resulting mappings, Bowtie returns more results with the caveat that they are more likely to be incorrect. The running time among the aligners varies within an order of magnitude with Bowtie consistently being the fastest while the slowest depended on the length and quality of the reads.

For the longer higher quality reads, the performance of BWA-PSSM lags slightly behind the other aligners for all PPV values (Additional file 1: Figure S2 and S3). These results are not unexpected as using quality values which are largely irrelevant should not improve the performance. Furthermore, some of the trade-offs employed to improve the performance for low-quality and biased reads impede the performance for high quality data. While disadvantageous, this is not the targeted type of data for which this approach was designed and the other aligners already serve this niche adequately.

GEM reports all the potential hits found and classifies them according to the edit distance from the genome. While this approach leads to a greater overall sensitivity, it also makes it difficult to assign a confidence value to a particular alignment. The strength of this mapper lies in aligning long insertion/deletion prone reads, two qualities which are conspicuously absent from the presented benchmark data sets. As such, the performance of GEM is presented in the data tables simply as a point of a comparison for this different class of read mappers.

For paired-end data (Table 2 and Additional file 1: Table S3), the situation is similar but more pronounced. Low quality reads are readily aligned with high accuracy by BWA-PSSM whereas high quality reads present a greater challenge. Due to the use of (nearly) default parameters for each aligner, BWA performs extremely poorly on the longer low quality reads. The situation is somewhat reversed for the high quality reads where BWA-PSSM finds slightly less hits in a longer amount of time than Bowtie2 and BWA. The results presented, of course, depend greatly upon the parameters chosen for each mapper (the default). Exploring the potential parameter space for each program is an overwhelming task which is often guided by the data to be aligned. The results presented here are merely meant to be a cross section of the potential capabilities of each aligner, corresponding to a roughly comparable (within an order of magnitude) running time.

### Ancient DNA

By specifying a probability for each base at each position, it is possible to include additional information in the alignment. Ancient DNA is fragmented and degraded in various ways, leading to specific biases and errors in the data. The dominant error is the deamination of cytosines into uracils (C-to-U), which will be interpreted as thymines in the sequencing step [40]. This leads to an excess of C-to-T or G-to-A mismatches, depending on the strand being sequenced. This is most significant in the ends of the reads and decreases rapidly towards the center [15].

PSSMs were simulated using the damage model specified by Orlando *et al.*[15], see Methods. The results (Table 3b) show that BWA-PSSM with a PSSM modelling the simulated damage gives slightly higher sensitivity and PPV than without a damage model. The sensitivity is slightly higher than BWA while the run time is roughly the same. BWA-PSSM was able to find more hits than BWA, Bowtie and Bowtie2 even without a specialized PSSM.

When mapping real ancient DNA and filtering on MapQ, the results mostly reflect the simulated data (Table 4a). The use of a PSSM led to the recovery of more matches than without one. While the difference is not large, the number of reads one might expect to be damaged is rather low in comparison to the total number of reads present. Hence, a modest increase in actual numbers can reflect a greater increase in relative terms. Furthermore, if the results reflect the simulated data, then the expected PPV of the filtered results should be higher than for the other mapping tools. Again, BWA-PSSM without a specialized PSSM provides an increase in filtered matches compared to BWA and Bowtie2.

**Table 4:** Analysis of real single-end data

		**Unfiltered**	**Filtered**	
	**Mapper**	**Aligned**	**Aligned**	**Time (s)**
		**(fraction)**	**(fraction)**	
**a) Ancient DNA**				
	BWA-PSSM^A^	0.01000	0.00763	60.75
	BWA-PSSM	0.01000	0.00759	51.61
	BWA	0.00906	0.00708	18.63
	BWA-MEM	0.01629	0.01177	21.24
	Bowtie	0.00892	*	63.78
	Bowtie2	0.01087	0.00691	16.48
	GEM	0.06106	*	18.69
**b) Real PAR-CLIP**				
	BWA-PSSM^PC^	0.600	0.148	146.08
	BWA-PSSM	0.476	0.114	141.08
	BWA	0.483	0.140	166.99
	BWA-MEM	0.198	0.022	236.51
	Bowtie	0.463	*	84.81
	Bowtie2	0.453	0.076	43.11
	GEM	0.227	0.227	280.57

Comparison of run times and fraction of reads mapped for the four mappers on two real data sets, each containing 100,000 Illumina reads. **a)** Reads of ancient DNA mapped to the horse genome. The run times do not include the time required to build the PSSMs. **b)** Reads of PAR-CLIP data mapped to the human genome. BWA-PSSM only makes use of the error model given by equation (1), while BWA-PSSM^A^ also uses the ancient DNA damage model and BWA-PSSM^PC^ also uses the T-to-C transition model characterizing PAR-CLIP data. The symbol ’*’ indicates that the mapper does not provide MapQ scores.

### PAR-CLIP data

Sequencing data from PAR-CLIP experiments is very prone to T-to-C transitions due to the incorporation of 4SU-containing nucleobases and their crosslinking to the bound protein. The locations of such transitions indicate where an RNA molecule is bound by a protein [41]. The increased probability of a T-to-C mismatch is readily encoded in a PSSM and incorporated into the mapping by BWA-PSSM.

**Figure 4:**
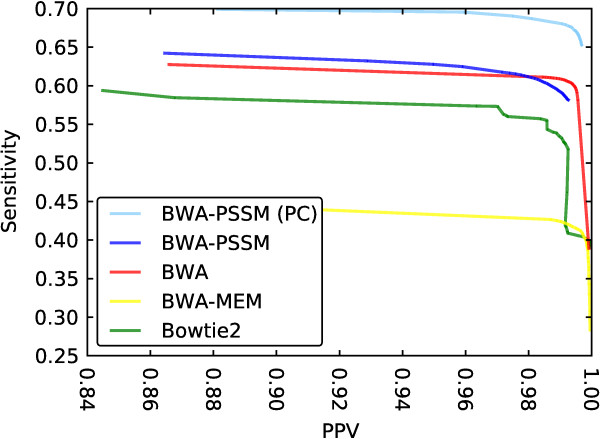
**Sensitivity as a function of PPV for BWA-PSSM, BWA, BWA-MEM and Bowtie2 using single-end PAR-CLIP data simulated using ART.** The curves for each mapping program were obtained by filtering for varying mapping qualities. The top line for BWA-PSSM (PC) includes the PAR-CLIP model. The results are based on the PAR-CLIP simulation shown in Table 3a. Bowtie and GEM are excluded as they do not provide MapQ scores.

To examine the efficacy of providing this extra information, reads were simulated with a 11% T-to-C rate. The results (Table 3a) show that the use of a T-to-C transition model improves both the unfiltered and filtered sensitivity. Without such a model, BWA-PSSM performs only slightly better than BWA. Introducing the error model increases the filtered sensitivity by nearly 14% over the previous best aligner (BWA) for quality-filtered mappings. This advantage, however, is not limited to high quality mappings and can be seen across all reported mapping qualities (Figure 4). Even for the high quality data sets, where the use of PSSM was more of a hindrance for aligning unbiased reads, introducing an error model leads to greatly improved sensitivity in aligning simulated PAR-CLIP data (Additional file 1: Table S2 and S4).

To support the simulated data, real data was obtained from a PAR-CLIP study investigating the binding of RNA to the HuR protein [42]. The statistics for the mapping of the real data (Table 4b) roughly reflects those for the simulated data. Notice the greatly reduced number of matches after filtering for all the mappers, which is the result of shorter reads leading to more ambiguous matches, and a base distribution unlike that of the actual genome (due to the AT-rich regions that were being sequenced). Nevertheless, BWA-PSSM finds more matches than any of the other programs at a PPV value greater than 0.95 for all types of simulated data.

### AT rich data

To test the effect of the background model in BWA-PSSM, we simulated Illumina reads of length 100nt using ART [43] from the *P. falciparum* genome [33], which has an AT content of more than 80%. In BWA-PSSM, the PSSMs were constructed using a background model, *q*(*g*), that matches the base composition of the reference genome, while the other mappers do not consider this base composition. We see that all mappers except BWA-MEM have similar PPVs for both unfiltered and filtered reads (Table 3c). Among the mappers that provide a quality score, BWA-PSSM obtains the highest sensitivity. For filtered reads BWA-MEM has the next highest sensitivity, however the PPV for BWA-MEM is significantly lower than for the other mappers. Bowtie2 has a marginally lower unfiltered sensitivity than BWA-PSSM, however, for the filtered reads the sensitivity of Bowtie2 is notably lower. While BWA has a considerably lower sensitivity than the other mappers, Bowtie has the highest sensitivity, which is obtained at the cost of slightly reduced PPV compared to the filtered PPV of BWA-PSSM.

### Random matches from contaminating reads

**Figure 5:**
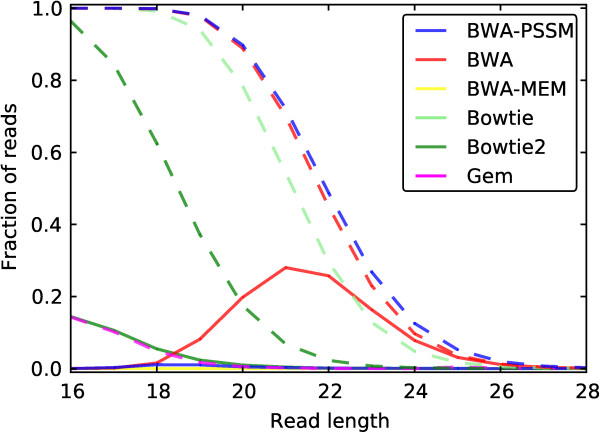
**Mapping simulated *E. coli * reads to the human genome.** The fraction of all *E. coli* reads that are mapped is shown as a function of the read length. For each length, 100,000 Illumina reads were simulated using ART [43]. The dotted lines represent the fraction of unfiltered reads, while the full lines are the fraction of quality filtered reads. For Bowtie and GEM curves are only shown for unfiltered reads. The curves for BWA-MEM are hardly visible as BWA-MEM does not map any reads.

We examined how well the mappers can filter out random short matches. To obtain random matches, we simulated short reads of different lengths from the *E. coli* genome [44] using ART [43] and mapped them to the human genome. From Figure 5 we observe that BWA-PSSM, BWA and Bowtie map similar fractions of reads when not applying a quality filter, while this fraction in general is smaller for Bowtie2. However, for quality filtered reads BWA-PSSM maps at worst less than 2% of the reads, which is less than any of the other mappers except BWA-MEM, though from read length 20nt the curves for Bowtie2 and BWA-PSSM are coincident. In comparison, BWA maps more than 25% of the reads at worst after quality filtering. GEM maps approximately the same fraction of reads as Bowtie2 does after quality filtering and BWA-MEM cannot map any reads at the given lengths. In conclusion, we see that BWA-PSSM has similar efficiency as BWA and Bowtie in mapping short reads, however the quality score (posterior probability) reported by BWA-PSSM allows for filtering out random matches at least as efficiently as Bowtie2.

### Xeno mapping

If no reference genome is available for a set of reads, one can try to map the reads to a closely related species. This task is called cross species mapping or xeno mapping [31]. Inspired by the work of Frith *et al.*[31], we examine how well the programs can map real reads from *D. melanogaster* using the closely related *D. simulans* genome as reference. We consider three sets of *D. melanogaster* reads obtained from the NCBI SRA database: a set of short reads (length 36nt, ID SRR001981), a set of long reads with low quality (length 76, ID SRR023647) and a set of long reads with high quality (length 95, ID SRR516029). All data sets are based on the Illumina platform and low quality bases in the end of the reads are trimmed using the AdapterRemoval tool [45].

To gauge the performance of the mappers, reads are mapped to both the *D. simulans* and *D. melanogaster* genome using each mapper. For each tool, we consider all reads that map to the genomes with a mapping quality above a given threshold, and we compare the mapping positions in the two genomes based on the UCSC whole genome alignment of *D. simulans* and *D. melanogaster*. If the mapping regions overlap in the whole genome alignment we say the read is *consistently* mapped. If the read maps to a region in *D. melanogaster* that is aligned to *D. simulans*, but the mapping regions do not overlap, we say the read is *inconsistently* mapped. For the xeno mapping experiments we calculate the sensitivity as the number of consistently mapped reads divided by the total number of reads, and the PPV is calculated as the number of consistently mapped reads divided by the number of consistently and inconsistently mapped reads.

The flexibility of BWA-PSSM allows us to include an evolutionary model that reflects the substitution level between the two Drosophila genomes when we map the reads to *D. simulans* (see Methods). For comparison we also map the reads to *D. simulans* with BWA-PSSM using only the standard error model. In both cases, we map the reads to *D. melanogaster* using only the standard error model.

**Figure 6:**
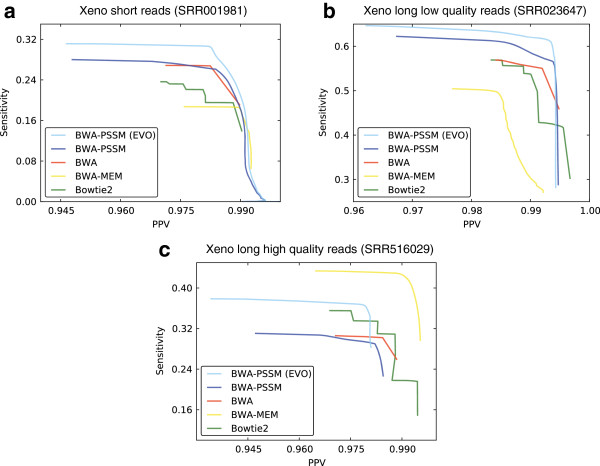
**Sensitivity as a function of PPV for BWA-PSSM, BWA, BWA-MEM and Bowtie2 for the xeno mapping experiment.** Single-end Illumina reads from *D. melanogaster* are mapped to the *D. simulans* genome using the five mappers. Plots are shown for short reads **(a)**, long low quality reads **(b)** and long high quality reads **(c)**. The curves for each mapping program were obtained by filtering for varying mapping qualities. The results are based on the data in Table 5. Bowtie and GEM are excluded as they do not provide MapQ scores.

Due to the setup of this experiment, we only consider mappers that provide MapQ scores. The results are shown in Table 5 and Figure 6. In Table 5 we used a MapQ threshold of 25 and in Figure 6 the threshold was varied between 0 and 200.

**Table 5:** Analysis of the xeno mapping experiment

		**MapQ filtered**		
	**Mapper**	**Sensitivity**	**PPV**	**Time (s)**
**a) Xeno short reads (SRR001981)**				
	BWA-PSSM^EVO^	0.305	0.982	3721.53
	BWA-PSSM	0.269	0.976	3228.94
	BWA	0.268	0.982	1444.48
	BWA-MEM	0.186	0.989	727.22
	Bowtie2	0.195	0.981	635.21
**b) Xeno long low quality reads (SRR023647)**				
	BWA-PSSM^EVO^	0.609	0.994	9355.64
	BWA-PSSM	0.591	0.989	7371.12
	BWA	0.550	0.992	3420.15
	BWA-MEM	0.500	0.984	1260.17
	Bowtie2	0.429	0.991	1262.93
**c) Xeno long high quality reads (SRR516029)**				
	BWA-PSSM^EVO^	0.364	0.979	13271.88
	BWA-PSSM	0.300	0.972	11457.96
	BWA	0.302	0.984	5735.58
	BWA-MEM	0.429	0.988	4147.98
	Bowtie2	0.218	0.987	3468.82

Comparison of sensitivity, positive predictive value (PPV) and run time using BWA-PSSM, BWA, BWA-MEM and Bowtie2 on real single-end reads from *D. melanogaster* mapped to *D. simulans*. In BWA-PSSM^EVO^ we include an evolutionary model reflecting the substitution rate between the two Drosophila genomes when mapping the reads to *D. simulans*, while in the BWA-PSSM results we only include the standard error model. The reported running time is only for the xeno mapping.

We see that BWA-PSSM with the evolutionary model generally performs better than all the other programs for the short reads and the long low quality reads (Figure 6a and 6b). BWA-PSSM has higher sensitivity for any PPV value except in the high PPV end of the curves. For the short reads, BWA-MEM has slightly better sensitivity at PPV between 0.991 and 0.992, and for the long low quality reads BWA and Bowtie2 can obtain slightly higher PPV than BWA-PSSM in the low sensitive end of the curves. For the long high quality reads (Figure 6c) we see that BWA-MEM performs best overall, while Bowtie2 can obtain marginally higher PPV than BWA-MEM. BWA-PSSM can obtain the next highest sensitivity with the evolutionary model, however all the other mappers, including BWA-PSSM without the evolutionary model, can obtain higher PPVs.

We see a similar picture when we consider the filtered results in Table 5. While the mappers have similar PPVs on all the data sets for a fixed MapQ threshold, BWA-PSSM has the highest sensitivity on the short and long low quality reads. On the long high quality reads BWA-MEM has the highest sensitivity and BWA-PSSM the next highest. We also see that the increased sensitivity of BWA-PSSM come at the cost of increased running time. In the worst case (Table 5b) BWA-PSSM is nearly 7.5 times slower than the fastest method (Bowtie2), however in this case BWA-PSSM maps over 40% more reads and have a marginal higher PPV than Bowtie2.

Finally we also see that the performance of BWA-PSSM generally improves when including an evolutionary model. On the short reads the sensitivity of BWA-PSSM is improved at all PPVs and on the two long read data sets BWA-PSSM can generally obtain higher sensitivity with the evolutionary model. However, BWA-PSSM can obtain slightly higher PPVs using only the standard error model on the long reads.

## Conclusions

We have presented BWA-PSSM, a novel method for using position-specific scoring matrices to provide quality aware short read mapping. The algorithm for PSSM scoring is based on BWA’s mapping algorithm. This method can be applied to other PSSM applications, lowering the number of genomic locations that need to be evaluated and increasing the efficiency of PSSM searches.

There are many advantages in the probabilistic approach taken here, in which the probability of a match being correct is estimated using prior probabilities that may be specific for the experiment. We have shown how it is possible to model the evolutionary differences between the sample and reference genome, sequencing errors based on e.g. quality scores, experiment-specific base substitutions, and contamination in the sample.

It is worth emphasizing the importance of the prior probability of a match. In all experiments there is a possibility that the read sequence is due to contamination or that it does not appear in the reference genome for other reasons (e.g. sequences private to the sampled individual). In some experiments the majority of reads may be contamination, as is often the case with ancient samples. Especially when it comes to short reads, there is a great risk that contaminating sequences will be mapped to the reference genome, as was illustrated with the mapping of *E. coli* sequences to the human genome. This may be taken into account by changing the value for the prior probability of a match.

Some of the important extensions implemented in BWA-PSSM include the ability to use longer genomes and to handle the forward and reverse search in a single concatenated index, the control of the direction of the search tree traversal based on the underlying PSSM, and the use of an interval heap for focusing on the most likely region of the search tree while discarding lower scoring branches. Furthermore, BWA-PSSM can capture platform and sample specific biases in the nucleotide composition, making the tool highly sensitive and adaptable to specialized applications such as ancient DNA, PAR-CLIP, or biased genomes.

Because matches can be prioritized by a continuous match score, the number of candidate matches stored in the heap can be significantly reduced compared to other methods that use the number of mismatches to prioritize. For this reason the PSSM implementation runs with a speed comparable to the other mappers.

## Methods

### A probabilistic model for next-generation DNA reads

In this section we present a detailed description of the probabilistic model of DNA reads. In the simple model we have two distinct processes that can affect the base called by the sequencer compared to the base actually present in the genome of interest: Actual mutation with respect to the reference genome from base *g* to base *a*, and a miscalled base *x* by the sequencing machine given base *a* in the sample.

**Figure 7:**
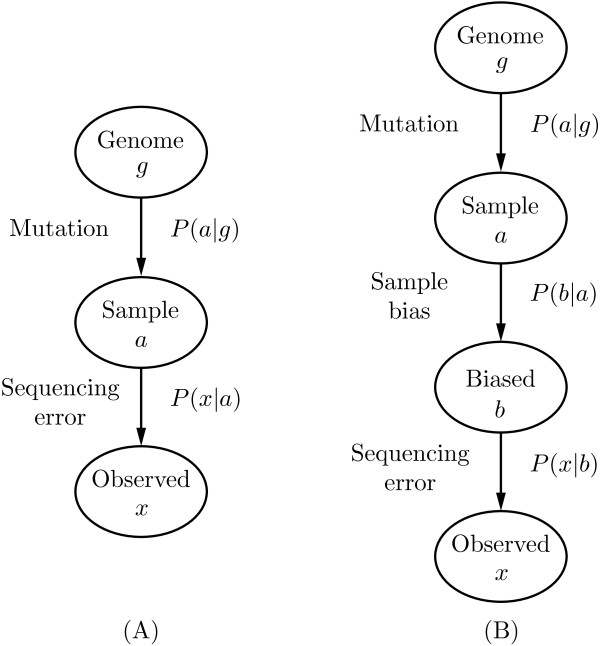
**Bayesian networks describing the independence assumptions between nucleotides.** The nodes represent random variables, while the arrows describe the conditional independencies between the variables. The left figure **(A)** shows the base in the reference genome *g*, the base in the sample *a*, and the observed base *x*, while the right figure **(B)** also includes the alternated (biased) base *b*.

We will assume that the observed base *x* is independent of the base *g* in the genome given the base *a* in the sample. This conditional independence assumption can be expressed by the Bayesian network in Figure 7A. Using this assumption we can calculate the probability of observing the base *x* in the read given the base *g* in the genome $P(x|g)=\sum_{a}P(x|a)P(a|g)$, where *P*(*a*|*g*) is the mutation model and *P*(*x*|*a*) is the model of sequencing errors. To calculate the entries in the position-specific scoring matrix (PSSM) we need *P*(*g*|*x*), and the conditional independence assumptions also give that

(3) $$P(g|x)=\underset{a}{\sum }P(g|a)P(a|x).$$

*P*(*g*|*a*) can be calculated from an evolutionary model. Here we use a simple one, where there is a fixed probability *p*_0_ for a mutation which is independent of the base, that is

(4) $$P(g|a)=\left\{\begin{array}{ll} 1-p_{0} & \text{if}\;a=g\\ p_{0}/3 & \text{if}\;a\neq g \end{array}\right)\;.$$

For this to make sense, we implicitly assume a uniform base distribution in the genome.

For sequencing error we also assume a simple model, where the probability of an error *p*_e_ = 10^-*Q*/10^ is given by the quality score *Q*. From this we can calculate the probability of the sample containing base *a* given that the sequencer is calling base *x*

(5) $$P(a|x)=\left\{\begin{array}{ll} 1-p_{e} & \text{if}\;a=x\\ p_{e}/3 & \text{if}\;a\neq x \end{array}\right)\;.$$

Note that it is in principle straight-forward to use more sophisticated models both for evolution and for sequencing errors and include these in the PSSM. An example of such a model is presented in the next section.

#### Evolutionary model for xeno mapping

For xeno mapping we adopt the evolutionary model by Frith *et al.*[31]. According to this model we write

$$P(g|a)=\left\{\begin{array}{ll} 1-p_{s} & \text{if}\;a=g\\ p_{s}p_{t} & \text{if}\;a\to g\;\text{is a transition}\\ \frac{1}{2}p_{s}(1-p_{t}) & \text{if}\;a\to g\;\text{is a transversion} \end{array}\right)\;,$$

where *p*_s_ is the probability of a substitution and *p*_t_ is the probability of a transition given that a substitution has happened. For mapping *D. melanogaster* reads to the *D. simulans* genome, we used the substitution and transition frequencies observed in the UCSC alignment of the two genomes as given by Frith *et al.*[31], that is ${\hat{p}}_{s}=0.15$ and ${\hat{p}}_{t}=0.45$.

### A probabilistic model for biased DNA reads

In a given data set, we might expect specific biases to affect the frequency with which particular modifications of the DNA sequence occur. This is a well-known phenomenon in many different types of data. If we include a specific model of these biases, we now have three processes that can affect the base called by the sequencer, where the additional process is changing the original base *a* into base *b* due to the type of DNA sample. The independence assumptions for these three processes is expressed by the Bayesian network in Figure 7B.

Using these assumptions the probability of genomic base *g* given that base *x* is reported by the sequencing machine is (as before)

(6) $$P(g|x)=\underset{a,b}{\sum }P(g|a)P(a|b)P(b|x),$$

where the term *P*(*a*|*b*) describes the bias. However, in the examples we will describe here, it is more natural to estimate *P*(*b*|*a*). By performing the *a*-sum in equation (6) and using the conditional independence assumptions, we obtain the expression

(7) $$P(g|x)=\sum_{b}P(g|b)P(b|x).$$

Using Bayes’ rule and the sum rule we can write the first term in the equation above as

(8) $$\begin{array}{ll} \;P(g|b) & =\frac{P(b|g)P(g)}{\sum_{g^{\prime }}P(b|g^{\prime })P(g^{\prime })}\;\text{where}\;P(b|g)\\ & =\underset{a}{\sum }P(b|a)P(a|g). \end{array}$$

Assuming the simple models for evolution *P*(*a*|*g*) and sequencing error *P*(*b*|*x*) from the previous section, all we need in order to calculate *P*(*g*|*x*) is an expression for the bias model *P*(*b*|*a*) and trivially a prior for the genomic base distribution *P*(*g*). In the following sections we will consider two such bias models.

#### Ancient DNA model

In ancient DNA we often observe damage (miscoding lesions), especially C-to-U changes due to deamination, which translate into C-to-T in the sequenced reads (or G-to-A depending on the strand being sequenced). The damage model *P*(*b*|*a*) describing these alterations is as follows. We allow C-to-T and G-to-A changes and the probabilities of these changes depend on the position in the read, with 3’ and 5’ positions having the highest rate of damage. Let *i* denote the position in the read, then we specify the probabilities *p*_*i*_(*b*|*a*) = 0 except for the following cases:

$$\begin{array}{ll} p_{i}(A|A)=1 & \;p_{i}(T|T)=1\\ p_{i}(T|C)=\gamma_{i} & \;p_{i}(C|C)=1-\gamma_{i}\\ p_{i}(A|G)=\delta_{i} & \;p_{i}(G|G)=1-\delta_{i}. \end{array}$$

The actual damage rates, *γ*_*i*_ and *δ*_*i*_, are obtained from Orlando *et al.*[15].

For ancient DNA, the evolutionary model *P*(*a*|*g*) is obtained by assuming a probability *m*_1_ for transitions (substitutions between C and T, or between A and G) and a smaller probability *m*_2_ for transversions (all other substitutions).

#### PAR CLIP model

While ancient DNA has a pattern of alterations where it is mainly bases in the 5’ and 3’ ends of a read that are modified, other types of data exhibit different modifications. PAR-CLIP data, for example, has consistent T-to-C changes. These changes can be handled in the same manner as for ancient DNA damage described above by modifying the conditional probabilities *P*(*b*|*a*) to construct a model *P*(*x*|*g*) incorporating the particular modification pattern.

#### Practical calculation of the PSSM

Once the evolutionary model, the bias model, and the error model have been formulated, *P*(*g*|*x*), and thus the corresponding PSSM scores, can be calculated for all possible combinations of base and quality score (and read position for the damage model). Therefore the conversion of a read with quality scores to a PSSM can be done very fast with a table look-up.

### Including indels in the alignment

In equation (2) we only considered alignments without indels. If we assume that the indel pattern **a** is independent of the starting position *ℓ* we can write the posterior probability for a match as

$$P(\ell ,a,M|x,g)=\frac{P(g|\ell ,a,M,x)P(a|M,x)P(\ell |M,x)P(M|x)}{P(g|M,x)P(M|x)+P(g|N,x)P(N|x)}.$$

Again we will assume that all unaligned positions in the genome are i.i.d. with the same distribution in both the match and mismatch model, which means that $P(g|\ell ,a,M,x)/P(g|N,x)=2^{S_{x}(\ell )}$. Consequently we can write the posterior probability as

$$P(\ell ,a,M|x,g)=\frac{2^{S_{x}(\ell )+\log_{2}P(a|M,x)}}{\sum_{\ell^{\prime },a^{\prime }}\;2^{S_{x}(\ell^{\prime })+\log_{2}\;P(a^{\prime }|M,x)}\;+\;L(1\;-\;P(M|x)\;)\;/\;P(M|x)},$$

where the sum in the denominator runs over all positions *ℓ*^′^ in the genome and all possible alignments **a**^′^ starting at position *ℓ*^′^.

For the indel pattern we will use a linear gap penalty model, which means that the gap lengths follow a geometric distribution [46]. If we assume that insertions and deletions happen independently at each position in **x**, we can write the probability of a given alignment in terms of the total number of insertions *a*_i_ and deletions *a*_d_ in the sequence

$$\begin{array}{ll} \;P(a|M,x) & =P(a_{i},a_{d}|M,x)\\ & =P(a_{i}|M,x)P(a_{d}|M,x)\propto 2^{-a_{i}\rho_{i}}2^{-a_{d}\rho_{d}}, \end{array}$$

where $p_{i}=2^{-\rho_{i}}$ is the probability of an insertion and $p_{d}=2^{-\rho_{d}}$ is the probability of a deletion. Here *ρ*_i_ and *ρ*_d_ are the insertion and deletion penalty log scores, respectively. In practice this is implemented as illustrated in Algorithm 1 and the default value for *ρ*_i_ and *ρ*_d_ is 17.

### Other BWA modifications

In addition to allowing the use of position-specific scoring matrices for alignment, we introduced two other modifications to speed up the alignment method and to allow for the indexing of larger genomes.

#### 64-Bit indexing

The existing version of BWA has been modified to use 64-bit indexing by changing the underlying data structures. This modification allows for the sequencing of genomes greater than 4 billion base pairs in length. Due to the limitations of the SAM/BAM format [47], however, these genomes must be composed of sub-sequences (i.e. chromosomes) smaller than 4 gigabases each. Using 64-bit indexing leads to an index approximately 32% larger than its 32-bit counterpart.

#### Single index

**Figure 8:**
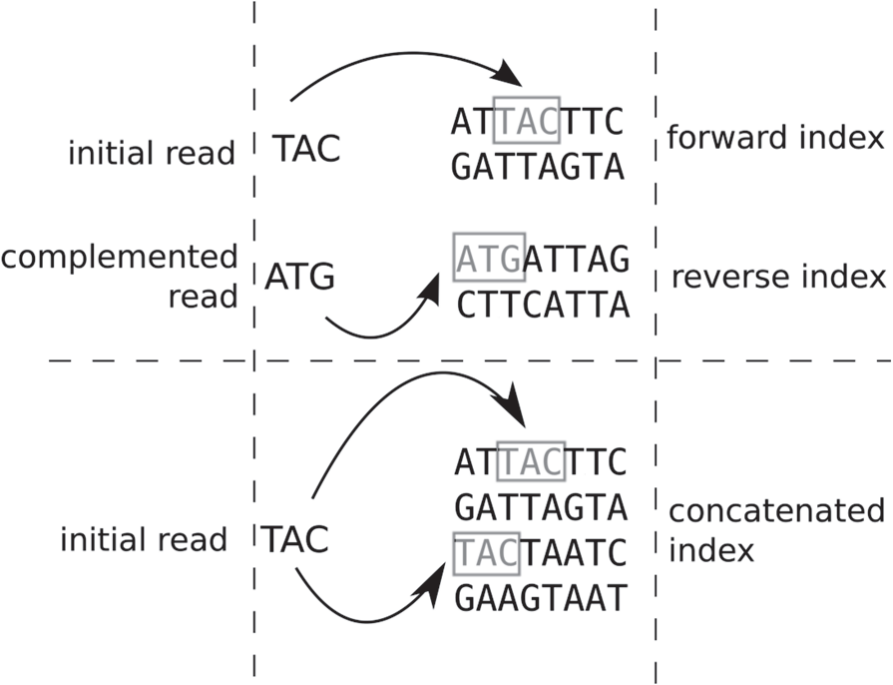
**Comparison of the original BWA alignment method (top) and the modified version using a single index (bottom).** The sequence of the short read, ‘TAC’, on the ‘-’ strand can easily be found by subtracting the length of the original sequence from its position in the concatenated sequence.

Traditionally, BWA uses two indexes: One of the forward and one of the reverse of the reference genome. Searches are performed by using the original read and a complemented read and searching the forward index and reverse index with the corresponding read. Combining the forward and reverse indexes into a single structure and searching using only the original read improves the running time by consolidating prefixes common to both the forward and reverse indexes (see Figure 8). Inspired by our work, a single 64-bit index has also been included in the 0.6 release of BWA.

#### Heap sorting for partial hits

In BWA, the best partial candidate hits are kept sorted by a score computed as a function of the mismatches, insertions and deletions in a heap-like structure. BWA-PSSM, in contrast, uses the offset from the best possible score at a particular position as the sorting criterion (Additional file 1: Figure S1). If the score of an alignment candidate (partial-hit) drops due to mismatches or insertions/deletions, it moves down the list. While BWA abandons the search when the size of the heap is exceeded, BWA-PSSM continues searching while discarding the partial hits with the lowest PSSM score offset. This is made possible by the use of an interval-heap data structure, allowing for the rapid removal, *Θ*(log n), of either the largest or smallest element [48].

The traditional breadth-first search of the prefix tree employed by BWA is further modified such that each branch is weighted according to the decrease from the optimal PSSM score for that character. That is, the branch corresponding to a perfectly matched base would have a weight corresponding to 0 since it will contain the best possible score (see Additional file 1: Figure S1). A mismatch branch, however, would have a lower score due to the use of a sub-optimal PSSM score. The use of score offsets corresponds to a breadth-first traversal of the suffix tree whereas using the total PSSM score itself corresponds to a depth-first traversal.

## Availability

BWA-PSSM and its source code are freely available through the website http://bwa-pssm.binf.ku.dk.

## Competing interests

The authors declare that they have no competing interests.

## Authors’ contributions

PK and AK developed the method and algorithms. JF and SL contributed to discussions on the method and algorithms. PK developed the software. PK, JF, SL, and AK designed the experiments and analysis. PK and JF performed the experiments and analysis. PK, JF, SL and AK wrote the manuscript. All authors read and approved the final manuscript.

## Supplementary Material

Additional file 1 Supplementary material. Click here for file
