# Supplementary material for: Adaptable probabilistic mapping of short reads using position specific scoring matrices
Source: BMC Bioinformatics. 2014 Apr 9;15:100. doi: 10.1186/1471-2105-15-100 (PMC4021105; doi:10.1186/1471-2105-15-100)
Supplement: Additional file 1 — Supplementary material. [file 1471-2105-15-100-S1.pdf]

Supplementary material for

Adaptable probabilistic mapping of short reads using position specific scoring matrices

Peter Kerpedjiev, Jes Frellsen, Stinus Lindgreen and Anders Krogh

1 Data sets

To assess the efficacy of PSSM-BWA in mapping various types of short reads, we created different sets of both real and simulated data. By including simulated data we can directly measure the sensitivity and PPV for each mapper.

The data sets are described below, except for the AT rich data, which was generated based on the *P. falciparum* genome (Pf3D7) version 2.1.5 obtained from the Sanger Institute (Gardner et al., 2002) using ART (Huang et al., 2012a), and the data set used for accessing random matches, which was generated from the *E. coli* genome (Blattner et al., 1997) with Genbank accession and version number U00096.2 using ART (Huang et al., 2012a).

1.1 Read Simulation

Reads of length 36, 50, 76, and 100 were simulated using the programs ART (Huang et al., 2012b), MASON (Holtgrewe, 2010) and WG-SIM (Li, 2011). The parameters used for each simulator are listed below:

ART Single End −f 0.01

ART Paired End −m 250 -s 60 -p -f 0.01

MASON Single End −N 100000

WG-SIM Single End −N 100000 -r 0.01 -S11 -d0 -e0

WG-SIM Paired End −N 100000 -r 0.001 -S11 -d250 -s50

1.2 Ancient DNA reads

Ancient DNA reads were simulated using 5' C-to-T and 3' G-to-A misincorporation rates estimated from (Orlando et al., 2011) and shown in Table S1. The template for the read lengths and quality scores was the single end length 55 simulated data described in the previous section. To saturate the data, only reads which contained simulated damage were kept for the alignment experiments. Real ancient DNA reads were obtained from the Illumina reads in the NCBI SRA data set SRP005902.

1.3 PAR-CLIP reads

Simulated reads were created by changing the base T to C at a rate of 0.11 in the single end length 36 simulated data set above. Real PAR-CLIP reads were obtained by sampling 100,000 reads from NCBI SRA data set SRR189777. Primers were removed from the reads using the AdapterRemoval tool (Lindgreen, 2012).

1.4 Xeno mapping

Three data sets were used for xeno mapping: a set of short reads (NCBI SRA data set SRR001981), a set of long reads with low quality (NCBI SRA data set ID SRR023647) and a set of long reads with high quality (NCBI SRA data setSRR516029). As reference genomes we used the April 2006 assembly of the *D. melanogaster* genome from the Berkeley Drosophila Genome Project and the April 2005 assembly of the *D. simulans* genome from the Genome Sequencing Center at Washington University School of Medicine in St. Louis. Both were obtained via the UCSC Genome Browser data downloads with IDs dm3 and droSim1. For comparing mapping positions in the two genomes we used the liftOver tool from the UCSC Genome Browser "kent" Bioinformatic Utilities and the associated liftOver data file (dm3ToDroSim1).

2 Mappers and Parameters

The performance of the program BWA-PSSM was tested on both simulated reads and real data from the Illumina platform. We compared the performance of BWA-PSSM to that of BWA (Li and Durbin, 2009), BWA-MEM (Li, 2013), Bowtie (Langmead et al., 2009), Bowtie2 (Langmead and Salzberg, 2012), and GEM (Marco-Sola et al., 2012) using the following options.

BWA-PSSM
- **Single End:** [no parameters]
- **Paired End** [no parameters]
- **Ancient DNA:** `-G error_model.txt`
- **PAR-CLIP:** `-G error_model.txt`

BWA
- **Single End:** [no parameters]
- **Paired End:** [no parameters]
- **Ancient DNA:** [no parameters]
- **PAR-CLIP:** [no parameters]

BWA-MEM
- **Single End:** [no parameters]
- **Paired End:** [no parameters]
- **Ancient DNA:** [no parameters]
- **PAR-CLIP:** [no parameters]

Bowtie
- **Single End:** `-y --best`
- **Paired End:** `-y --best`
- **Ancient DNA:** `-y --best`
- **PAR-CLIP:** `-y --best`

Bowtie2
- **Single End:** `--very-sensitive`
- **Paired End:** `--very-sensitive`

Ancient DNA: `--very-sensitive`

PAR-CLIP: `--very-sensitive`

GEM

Single End: `-q offset-33 --unique-mapping`

Paired End: `-q offset-33 --unique-mapping`

Ancient DNA: `-q offset-33 --unique-mapping`

PAR-CLIP: `-q offset-33 --unique-mapping`

In all of the tests, comparisons are shown between the raw alignment as well as the quality filtered alignment. The quality filtered alignment discards all mappings with a MapQ of less than 25.

2.1 Mapping ancient DNA with BWA-PSSM

The ancient DNA reads were converted to a PSSM using the provided fastq2wm33.pl script using the following command.

```
cat reads.fastq | ./fastq2wm33.pl > reads.pssm
```

The BWA-PSSM mapper was then invoked as usual except instead of passing the fastq file as a parameter, the PSSM was used.

3 Mapping probabilities for ungapped alignments

In this section we will derive the mapping match probability $P(\ell, \mathrm{M}|\mathbf{x}, \mathbf{g})$, which is the probability of the alignment position ℓ and the foreground model M given a read $\mathbf{x}$ and the genome $\mathbf{g}$. Here we assume that the read is aligned to starting position ℓ in the genome using only end-gaps in the read sequence and no gaps in the genome.

Using the sum and product rules we can express the match probability as

$$\begin{aligned} P(\ell, \mathrm{M}|\mathbf{x}, \mathbf{g}) &= \frac{P(\ell, \mathrm{M}, \mathbf{g}|\mathbf{x})}{P(\mathbf{g}|\mathbf{x})} \\ &= \frac{P(\mathbf{g}|\ell, \mathrm{M}, \mathbf{x})P(\ell|\mathrm{M}, \mathbf{x})P(\mathrm{M}|\mathbf{x})}{P(\mathbf{g}|\mathbf{x})} \\ &= \frac{P(\mathbf{g}|\ell, \mathrm{M}, \mathbf{x})P(\ell|\mathrm{M}, \mathbf{x})P(\mathrm{M}|\mathbf{x})}{P(\mathbf{g}|\mathrm{M}, \mathbf{x})P(\mathrm{M}|\mathbf{x}) + P(\mathbf{g}|\mathrm{N}, \mathbf{x})P(\mathrm{N}|\mathbf{x})} \\ &= \frac{P(\mathbf{g}|\ell, \mathrm{M}, \mathbf{x})P(\ell|\mathrm{M}, \mathbf{x})}{P(\mathbf{g}|\mathrm{M}, \mathbf{x}) + P(\mathbf{g}|\mathrm{N}, \mathbf{x})P(\mathrm{N}|\mathbf{x})/P(\mathrm{M}|\mathbf{x})} \\ &= \frac{P(\mathbf{g}|\ell, \mathrm{M}, \mathbf{x})P(\ell|\mathrm{M}, \mathbf{x})}{P(\mathbf{g}|\mathrm{M}, \mathbf{x}) + P(\mathbf{g}|\mathrm{N}, \mathbf{x})(1 - P(\mathrm{M}|\mathbf{x}))/P(\mathrm{M}|\mathbf{x})}, \end{aligned}$$

where we used that $P(\mathrm{N}|\mathbf{x}) + P(\mathrm{M}|\mathbf{x}) = 1$. Using the sum and product rule, we can write

$$P(\mathbf{g}|\mathrm{M}, \mathbf{x}) = \sum_{\ell'} P(\mathbf{g}|\ell', \mathrm{M}, \mathbf{x})P(\ell'|\mathrm{M}, \mathbf{x})\,. \quad (1)$$

If we assume that all mapping positions are equally likely *a priori* we have that $P(\ell|\mathrm{M},\mathbf{x}) = 1/L$, where $L = |\mathbf{g}|$ is the length of the genome. Using this assumption and equation (1) we get

$$\begin{aligned}
P(\ell,\mathrm{M}|\mathbf{x},\mathbf{g}) &= \frac{P(\mathbf{g}|\ell,\mathrm{M},\mathbf{x})P(\ell|\mathrm{M},\mathbf{x})}{\sum_{\ell'} P(\mathbf{g}|\ell',\mathrm{M},\mathbf{x})P(\ell'|\mathrm{M},\mathbf{x}) + P(\mathbf{g}|\mathrm{N},\mathbf{x})(1-P(\mathrm{M}|\mathbf{x}))/P(\mathrm{M}|\mathbf{x})} \\
&= \frac{P(\mathbf{g}|\ell,\mathrm{M},\mathbf{x})}{\sum_{\ell'} P(\mathbf{g}|\ell',\mathrm{M},\mathbf{x}) + LP(\mathbf{g}|\mathrm{N},\mathbf{x})(1-P(\mathrm{M}|\mathbf{x}))/P(\mathrm{M}|\mathbf{x})} \\
&= \frac{P(\mathbf{g}|\ell,\mathrm{M},\mathbf{x})/P(\mathbf{g}|\mathrm{N},\mathbf{x})}{\sum_{\ell'} P(\mathbf{g}|\ell',\mathrm{M},\mathbf{x})/P(\mathbf{g}|\mathrm{N},\mathbf{x}) + L(1-P(\mathrm{M}|\mathbf{x}))/P(\mathrm{M}|\mathbf{x})}\,.
\end{aligned}$$

We will assume that the bases in the genome are independent both in the foreground and background model. In the background model N we will also assume that the genome is independent of the read, which means that we can write

$$P(\mathbf{g}|\mathrm{N},\mathbf{x}) = P(\mathbf{g}|\mathrm{N}) = \prod_{i=1}^{|\mathbf{g}|} P(g_i|\mathrm{N})\,. \tag{2}$$

In the foreground model M, we will assume that (a) the i'th genome base is independent of all read bases except the read base it is aligned to. Furthermore we will assume that (b) the probability of an unaligned genome base in the foreground model is the same as in the background model. Finally we will assume that (c) the probability of an aligned genome base is independent of the starting position of the alignment ℓ given the aligned read base. Based on these three assumptions we can write

$$\begin{aligned}
P(\mathbf{g}|\ell,\mathrm{M},\mathbf{x}) &= \prod_{i=1}^{|\mathbf{g}|} P(g_i|\ell,\mathrm{M},\mathbf{x}) \\
&= \prod_{i=1}^{\ell-1} P(g_i|\ell,\mathrm{M},\mathbf{x}) \prod_{i=\ell}^{\ell+|\mathbf{x}|-1} P(g_i|\ell,\mathrm{M},\mathbf{x}) \prod_{i=\ell+|\mathbf{x}|}^{|\mathbf{g}|} P(g_i|\ell,\mathrm{M},\mathbf{x}) \\
&\overset{(a)}{=} \prod_{i=1}^{\ell-1} P(g_i|\ell,\mathrm{M}) \prod_{i=\ell}^{\ell+|\mathbf{x}|-1} P(g_i|\ell,\mathrm{M},x_{i-\ell+1}) \prod_{i=\ell+|\mathbf{x}|}^{|\mathbf{g}|} P(g_i|\ell,\mathrm{M}) \\
&\overset{(b)}{=} \prod_{i=1}^{\ell-1} P(g_i|\mathrm{N}) \prod_{i=\ell}^{\ell+|\mathbf{x}|-1} P(g_i|\ell,\mathrm{M},x_{i-\ell+1}) \prod_{i=\ell+|\mathbf{x}|}^{|\mathbf{g}|} P(g_i|\mathrm{N}) \\
&\overset{(c)}{=} \prod_{i=1}^{\ell-1} P(g_i|\mathrm{N}) \prod_{i=\ell}^{\ell+|\mathbf{x}|-1} P(g_i|\mathrm{M},x_{i-\ell+1}) \prod_{i=\ell+|\mathbf{x}|}^{|\mathbf{g}|} P(g_i|\mathrm{N})\,.
\end{aligned} \tag{3}$$

Using equations (2) and (3) we can write the ratio of the genome probability according to the foreground model and background model as

$$\begin{aligned}
\frac{P(\mathbf{g}|\ell,\mathrm{M},\mathbf{x})}{P(\mathbf{g}|\mathrm{N},\mathbf{x})} &= \frac{\prod_{i=1}^{\ell-1} P(g_i|\mathrm{N}) \prod_{i=\ell}^{\ell+|\mathbf{x}|-1} P(g_i|\mathrm{M},x_{i-\ell+1}) \prod_{i=\ell+|\mathbf{x}|}^{|\mathbf{g}|} P(g_i|\mathrm{N})}{\prod_{i=1}^{|\mathbf{g}|} P(g_i|\mathrm{N})} \\
&= \prod_{i=\ell}^{\ell+|\mathbf{x}|-1} \frac{P(g_i|\mathrm{M},x_{i-\ell+1})}{P(g_i|\mathrm{N})}
\end{aligned} \tag{4}$$

We then define the log-odds score $S(\mathbf{g}|\ell,\mathbf{x}) \stackrel{\text{def}}{=} \log_2 \frac{P(\mathbf{g}|\ell,\mathrm{M},\mathbf{x})}{P(\mathbf{g}|\mathrm{N},\mathbf{x})}$, which based on equation (4) can be written as

$$S(\mathbf{g}|\ell,\mathbf{x}) = \sum_{i=\ell}^{\ell+|\mathbf{x}|-1} S(g_i|x_{i-\ell+1}) = \sum_{j=1}^{|\mathbf{x}|} S(g_{\ell+j-1}|x_j)\,, \tag{5}$$

where $S(g_{\ell+j-1}|x_j) = \log_2 \frac{P(g_{\ell+j-1}|\mathrm{M},x_j)}{P(g_{\ell+j-1}|\mathrm{N})}$. Clearly the individual log-odds scores

$$\{S(\gamma|x_k)\}_{\gamma\in\{\mathtt{A},\mathtt{C},\mathtt{G},\mathtt{T}\},k=1,\ldots,|\mathbf{x}|}$$

can naturally be represented as a PSSM and from equation (5) we see that $S(\mathbf{g}|\ell,\mathbf{x})$ can be calculated by scoring the sequence $g_{\ell:\ell+|\mathbf{x}|}$ with this matrix.

So finally we can express the mapping match probability in terms of PSSM scores by

$$P(\ell,\mathrm{M}|\mathbf{x},\mathbf{g}) = \frac{2^{S(\mathbf{g}|\ell,\mathbf{x})}}{\sum_{\ell'} 2^{S(\mathbf{g}|\ell',\mathbf{x})} + L(1-P(\mathrm{M}|\mathbf{x}))/P(\mathrm{M}|\mathbf{x})}\,,$$

which is the result given in the paper.

References

Blattner FR, Plunkett G, Bloch CA, Perna NT, Burland V, Riley M, Collado-Vides J, Glasner JD, Rode CK, Mayhew GF, et al.. 1997. The complete genome sequence of escherichia coli k-12. *Science* **277**: 1453–1462.

Gardner MJ, Hall N, Fung E, White O, Berriman M, Hyman RW, Carlton JM, Pain A, Nelson KE, Bowman S, et al.. 2002. Genome sequence of the human malaria parasite Plasmodium falciparum. *Nature* **419**: 498–511.

Holtgrewe M. 2010. Mason–a read simulator for second generation sequencing data. *Technical Report FU Berlin* .

Huang W, Li L, Myers J, and Marth G. 2012a. ART: a next-generation sequencing read simulator. *Bioinformatics* **28**: 593–4.

Huang W, Li L, Myers JR, and Marth GT. 2012b. ART: a next-generation sequencing read simulator. *Bioinformatics (Oxford, England)* **28**: 593–594.

Langmead B and Salzberg SL. 2012. Fast gapped-read alignment with Bowtie 2. *Nature Methods* **9**: 357–359.

Langmead B, Trapnell C, Pop M, and Salzberg S. 2009. Ultrafast and memory-efficient alignment of short DNA sequences to the human genome. *Genome Biology* **10**: R25+.

Li H. 2011. wgsim - Read simulator for next generation sequencing.

Li H. 2013. Aligning sequence reads, clone sequences and assembly contigs with BWA-MEM. *ArXiv e-prints* .

Li H and Durbin R. 2009. Fast and accurate short read alignment with Burrows-Wheeler transform. *Bioinformatics (Oxford, England)* **25**: 1754–1760.

Lindgreen S. 2012. Adapterremoval: Easy cleaning of next generation sequencing reads. *BMC Research Notes* **5**: 337.

Marco-Sola S, Sammeth M, Guigo R, and Ribeca P. 2012. The GEM mapper: fast, accurate and versatile alignment by filtration. *Nat Meth* **9**: 1185–1188.

Orlando L, Ginolhac A, Raghavan M, Vilstrup J, Rasmussen M, Magnussen K, Steinmann KE, Kapranov P, Thompson JF, Zazula G, et al.. 2011. True single-molecule DNA sequencing of a pleistocene horse bone. *Genome Research* **21**: 1705–1719.

PSSM

	1	2	3
**A**	-2	-5	-5
**C**	-4	-7	-2
**G**	2	-7	-5
**T**	-5	2	1

Offsets

	1	2	3
**A**	-4	-7	-6
**C**	-6	-9	-3
**G**	0	-9	-6
**T**	-7	0	0

Cropped Suffix Tree

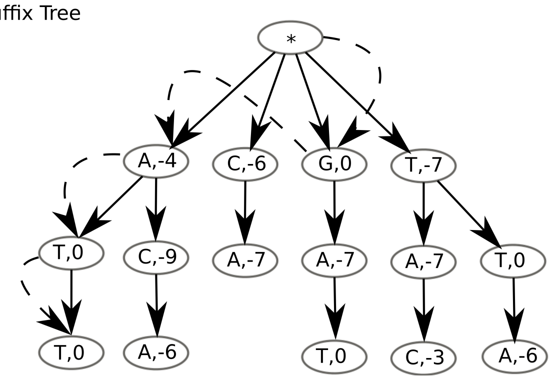

Figure S1: The search path for BWA-PSSM. The PSSM is converted into a set of offsets from the maximum score as shown in the top. Below, the partial prefix tree to depth three is shown for the sequence GATTACA. To search for matches of the PSSM in the sequence, partial hits are scored according to the greatest offset encountered so far. In the illustrated search, the highest scoring string would be ’GTT’. After first visiting the ’G’, there is no ’T’ on that branch and the ’A’ in the next position would lead to a combined offset of -7. Thus, the value of ’A’ in the first position (with a score offset of -4) will rise to the top of the heap and be visited next.

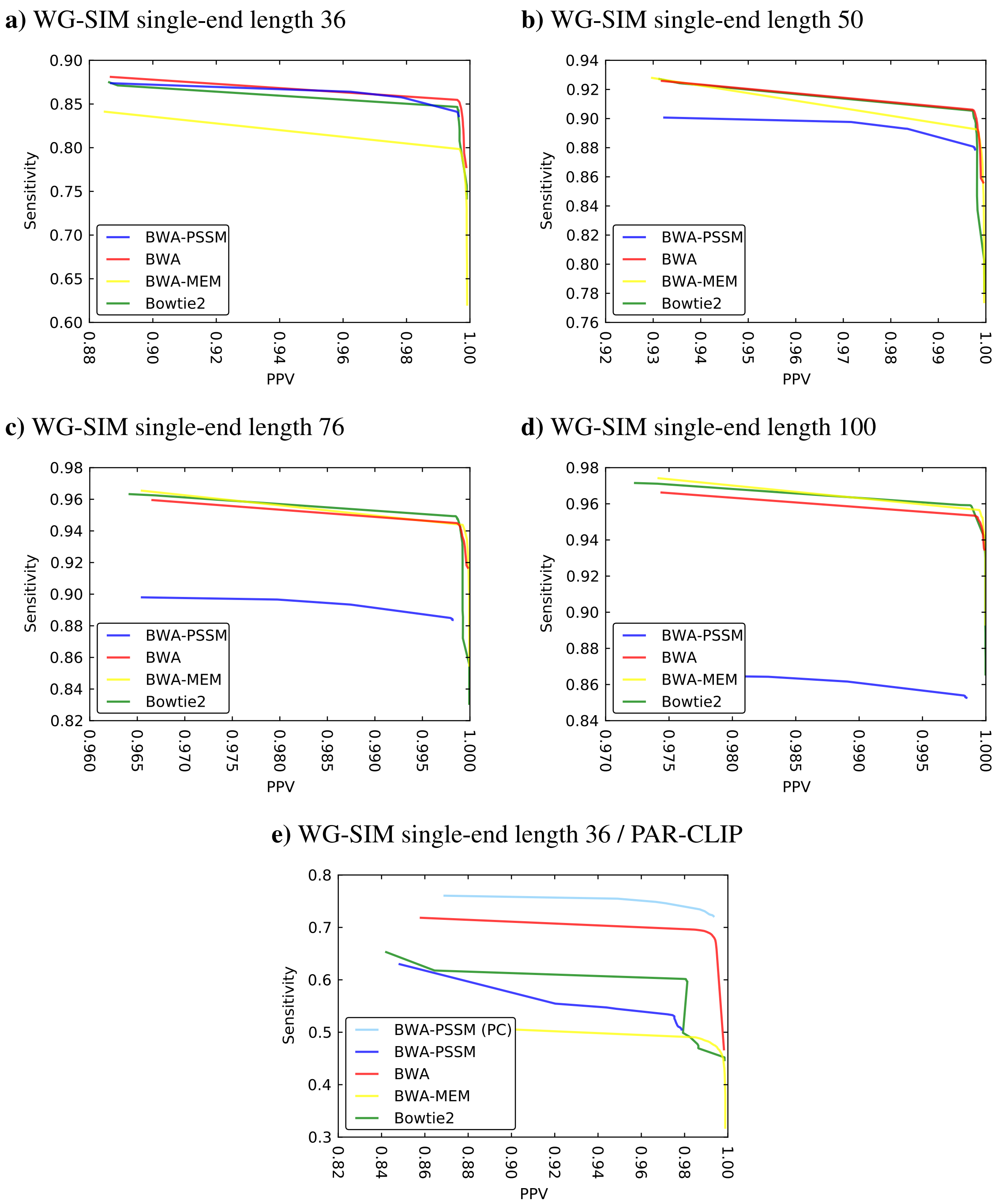

Figure S2: *Sensitivity as a function of PPV for BWA-PSSM, BWA, BWA-MEM and Bowtie2 using single-end WG-SIM-simulated data.* Curves are shown for reads of length 36, 50, 76, 100 and reads of length 36 with simulated mutations corresponding to a PAR-CLIP experiment. The curves for each mapping program were obtained by filtering for varying mapping qualities. The results are based on the simulations shown in Table S2. Bowtie and GEM are excluded as they do not provided MapQ scores.

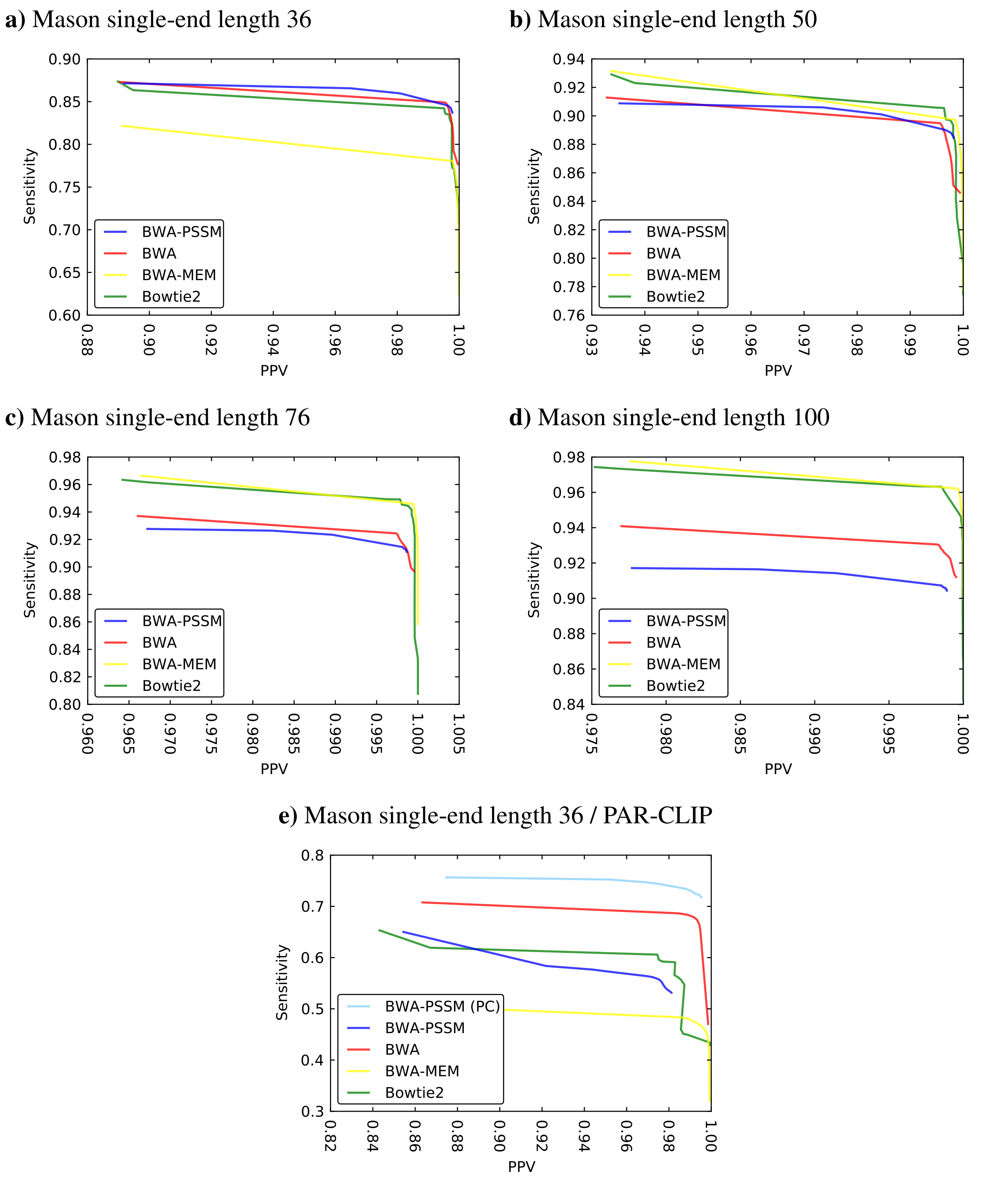

Figure S3: *Sensitivity as a function of PPV for BWA-PSSM, BWA, BWA-MEM and Bowtie2 using single-end MASON-simulated data.* Curves are shown for reads of length 36, 50, 76, 100 and reads of length 36 with simulated mutations corresponding to a PAR-CLIP experiment. The curves for each mapping program were obtained by filtering for varying mapping qualities. The results are based on the simulations shown in Table S4.Bowtie and GEM are excluded as they do not provided MapQ scores.

Damage	Position					
	0	1	2	3	4	5
5' C→T	0.307	0.160	0.067	0.043	0.032	0.024
3' G→A	0.307	0.160	0.067	0.043	0.032	0.024

Table S1: The base misincorporation rates used for simulating ancient DNA reads. These same rates are used in constructing the PSSMs for alignment. The 3' damage positions are offsets from the last position (0 refers to the last read position, 1 refers to the second to last position) while 5' damage positions are offsets from the beginning of the read.

Mapper	Unfiltered Sensitivity	Unfiltered PPV	MapQ filtered Sensitivity	MapQ filtered PPV	Time (s)
**a) WG-SIM single-end length 36**					
BWA-PSSM	0.874	0.887	0.838	0.996	41.24
BWA	0.881	0.887	0.794	0.998	52.56
BWA-MEM	0.841	0.885	0.720	0.999	209.11
Bowtie	0.860	0.884	*	*	14.75
Bowtie2	0.875	0.886	0.808	0.997	32.35
GEM	0.827	0.996	*	*	37.13
**b) WG-SIM single-end length 50**					
BWA-PSSM	0.901	0.932	0.880	0.998	55.67
BWA	0.926	0.932	0.859	0.999	64.46
BWA-MEM	0.928	0.930	0.818	1.000	89.98
Bowtie	0.891	0.932	*	*	19.84
Bowtie2	0.927	0.931	0.847	0.998	52.66
GEM	0.879	0.998	*	*	31.65
**c) WG-SIM single-end length 76**					
BWA-PSSM	0.898	0.965	0.884	0.998	78.06
BWA	0.960	0.967	0.918	1.000	83.23
BWA-MEM	0.965	0.965	0.890	1.000	47.65
Bowtie	0.892	0.966	*	*	31.37
Bowtie2	0.963	0.964	0.890	0.999	107.37
GEM	0.913	0.999	*	*	31.61
**d) WG-SIM single-end length 100**					
BWA-PSSM	0.865	0.974	0.853	0.998	98.18
BWA	0.966	0.974	0.935	1.000	104.08
BWA-MEM	0.974	0.974	0.916	1.000	47.25
Bowtie	0.868	0.974	*	*	42.29
Bowtie2	0.972	0.972	0.912	1.000	140.51
GEM	0.921	1.000	*	*	39.65
**e) WG-SIM single-end length 36 / PAR-CLIP**					
BWA-PSSM^{PC}	0.760	0.869	0.724	0.992	60.38
BWA-PSSM	0.630	0.848	0.512	0.977	80.96
BWA	0.718	0.858	0.659	0.995	61.66
BWA-MEM	0.518	0.832	0.443	0.998	101.24
Bowtie	0.706	0.857	*	*	38.59
Bowtie2	0.653	0.842	0.470	0.986	28.31
GEM	0.496	0.979	*	*	44.08
**f) WG-SIM single-end length 76 / Ancient DNA**					
BWA-PSSM^{A}	0.895	0.965	0.880	0.998	97.67
BWA-PSSM	0.852	0.964	0.837	0.997	96.13
BWA	0.953	0.965	0.913	1.000	98.32
BWA-MEM	0.965	0.965	0.888	1.000	47.61
Bowtie	0.871	0.964	*	*	37.87
Bowtie2	0.961	0.962	0.851	0.999	106.99
GEM	0.905	0.999	*	*	33.40

Table S2: *Analysis of single-end data simulated with WG-SIM.* Comparison of sensitivity, positive predictive value (PPV) and run time using BWA-PSSM, BWA, BWA-MEM, Bowtie, Bowtie2 and GEM on simulated data sets covering a random 1% of the human genome. The reads were simulated using the WG-SIM (Li, 2011) program with the parameters listed in the Read Simulation section.

	Unfiltered		MapQ filtered		
Mapper	Sensitivity	PPV	Sensitivity	PPV	Time (s)
**a) WG-SIM paired-end length 36**					
BWA-PSSM	0.953	0.955	0.888	0.999	287.35
BWA	0.958	0.959	0.880	1.000	231.67
BWA-MEM	0.855	0.877	0.639	1.000	284.86
Bowtie	0.458	0.918	*	*	1407.87
Bowtie2	0.946	0.954	0.845	0.999	120.36
GEM	0.921	0.090	*	*	95.43
**b) WG-SIM paired-end length 50**					
BWA-PSSM	0.961	0.964	0.918	0.999	238.18
BWA	0.971	0.973	0.922	1.000	182.54
BWA-MEM	0.926	0.927	0.811	1.000	156.47
Bowtie	0.454	0.952	*	*	553.43
Bowtie2	0.968	0.969	0.874	1.000	140.81
GEM	0.930	0.319	*	*	61.87
**c) WG-SIM paired-end length 76**					
BWA-PSSM	0.959	0.969	0.933	0.997	282.65
BWA	0.980	0.981	0.948	1.000	213.00
BWA-MEM	0.964	0.964	0.883	1.000	126.64
Bowtie	0.414	0.974	*	*	390.69
Bowtie2	0.978	0.978	0.886	1.000	183.93
GEM	0.942	0.454	*	*	50.20
**d) WG-SIM paired-end length 100**					
BWA-PSSM	0.949	0.969	0.930	0.996	339.30
BWA	0.983	0.985	0.958	1.000	292.41
BWA-MEM	0.974	0.974	0.912	1.000	150.41
Bowtie	0.349	0.981	*	*	420.18
Bowtie2	0.981	0.981	0.893	1.000	218.15
GEM	0.946	0.476	*	*	51.51

Table S3: *Analysis of paired-end data simulated with WG-SIM.* Comparison of sensitivity, positive predictive value (PPV) and run time using BWA-PSSM, BWA, BWA-MEM, Bowtie, Bowtie2 and GEM on simulated data sets covering 1% of the human genome. The reads were simulated using the WG-SIM (Li, 2011) program with the parameters listed in the Read Simulation section. Insert sizes in the paired-end data were simulated using a mean length of 250 and a standard deviation of 50.

Mapper	Unfiltered Sensitivity	Unfiltered PPV	MapQ filtered Sensitivity	MapQ filtered PPV	Time (s)
**a) Mason single-end length 36**					
BWA-PSSM	0.872	0.892	0.841	0.997	40.64
BWA	0.873	0.890	0.793	0.998	54.68
BWA-MEM	0.822	0.891	0.706	1.000	195.39
Bowtie	0.823	0.887	*	*	19.58
Bowtie2	0.874	0.890	0.778	0.998	31.98
GEM	0.823	0.997	*	*	39.99
**b) Mason single-end length 50**					
BWA-PSSM	0.909	0.935	0.887	0.998	53.85
BWA	0.913	0.933	0.851	0.998	74.30
BWA-MEM	0.932	0.934	0.823	1.000	99.54
Bowtie	0.829	0.931	*	*	29.48
Bowtie2	0.929	0.934	0.841	0.999	52.37
GEM	0.870	0.998	*	*	33.68
**c) Mason single-end length 76**					
BWA-PSSM	0.928	0.967	0.912	0.999	75.16
BWA	0.937	0.966	0.899	0.999	105.91
BWA-MEM	0.967	0.967	0.893	1.000	56.05
Bowtie	0.797	0.966	*	*	51.83
Bowtie2	0.963	0.964	0.870	1.000	104.54
GEM	0.898	0.999	*	*	34.29
**d) Mason single-end length 100**					
BWA-PSSM	0.917	0.978	0.905	0.999	94.74
BWA	0.941	0.977	0.914	0.999	157.19
BWA-MEM	0.978	0.978	0.922	1.000	51.94
Bowtie	0.751	0.977	*	*	73.98
Bowtie2	0.974	0.975	0.891	1.000	140.96
GEM	0.912	1.000	*	*	42.26
**e) Mason single-end length 36 / PAR-CLIP**					
BWA-PSSM^{PC}	0.757	0.875	0.722	0.994	61.45
BWA-PSSM	0.650	0.854	0.540	0.978	80.95
BWA	0.708	0.863	0.652	0.995	61.51
BWA-MEM	0.509	0.840	0.438	0.998	98.63
Bowtie	0.681	0.860	*	*	40.76
Bowtie2	0.653	0.843	0.459	0.986	28.11
GEM	0.495	0.981	*	*	45.59
**f) Mason single-end length 76 / Ancient DNA**					
BWA-PSSM^{A}	0.917	0.966	0.901	0.998	97.38
BWA-PSSM	0.899	0.965	0.883	0.997	93.74
BWA	0.931	0.964	0.894	0.999	120.74
BWA-MEM	0.966	0.966	0.891	1.000	56.12
Bowtie	0.784	0.964	*	*	55.35
Bowtie2	0.961	0.962	0.833	0.999	106.46
GEM	0.891	0.999	*	*	36.21

Table S4: *Analysis of single-end data simulated with MASON.* Comparison of sensitivity, positive predictive value (PPV) and run time using BWA-PSSM, BWA, BWA-MEM, Bowtie, Bowtie2 and GEM on simulated data sets covering a random 1% of the human genome. The reads were simulated using the MASON (Holtgrewe, 2010) program with the parameters listed in the Read Simulation section.
